# Differing Systems of Patients' Contributions

**Published:** 1913-12-13

**Authors:** 


### DIFFERING SYSTEMS OF PATIENTS' CONTRIBUTIONS.

The question of how much a patient who is not truly necessitous should pay towards the cost of his maintenance while in hospital is one which should engage the attention of all interested in hospital administration. Many schemes are in practice, but a standard system of inquiry and payment would be of great use. One hospital may accept weekly payments of a guinea or half a guinea; another institution may expect a donation of gratitude at the conclusion of treatment; a third will be content with drawing the patient's attention to the ward collecting-box; while a fourth will not admit cases who are not considered to be of the class for which the particular institution is intended.

Of the four systems, the one of a donation at the end of the treatment is perhaps the best. A patient who is paying even a small sum weekly is apt to regard himself as a little above the other cases in the ward, and will often expect privileges, such as extra visitors, to which he considers himself entitled by reason of the payment. The cases under treatment in hospitals are admitted through three channels. Some come by way of the out-patient department, others are sent in by general practitioners and members of the staff, and the remainder are casualties requiring immediate attention. The almoners can deal effectually with the admissions from the out-patient department, and there is usually plenty of time before the day of admission to make inquiries to substantiate a patient's statement as to his means and consequent ability or inability to contribute. The cases sent in by outside doctors are the ones which require investigation. In many cases the medical man who sends up the case will tell his patient that a donation is expected, and very often he will be able to suggest an amount. With other cases it is not so easy to introduce the subject of contribution. Many urgent cases who are sent in by outside doctors are quite able to pay the fees of a nursing home, although this fact may not be known to the doctor. In a case of this nature it is most important that the secretary should see the friends of the patient at the earliest opportunity, and make an arrangement whereby the hospital receives a good donation. Another type of case which should be followed up is the patient who is admitted from an institution or a home belonging to a public body. Unions in the country often send up patients requiring treatment, and if approached the Guardians will sometimes make the hospital a substantial grant. The names of workmen who come within the scope of the Workmen's Compensation Act, and who have received injury at work, should be noted, and at the conclusion of the treatment a letter should be addressed to the employers.

It may also be added that street accidents often lead to handsome donations. A child may be run over by a motor-car and seriously injured, requiring a considerable stay in hospital and subsequent convalescence. The hall porter should obtain the address of the owner of the car, and very often, although the chauffeur may not be legally responsible for the accident, his employer may be willing to send a cheque in recognition of the treatment received. The employers of servants will very often acknowledge in a practical manner the benefit their employees have received if the matter is brought to their notice. A casual stroll through the wards on visiting days will occasionally show the secretary several patients whose relatives have a well-to-do appearance. By daily watching the admission book a secretary will find cases which will lead to donations.

